# *catena*-Poly[[bis­(4-methyl­benzene­thiol­ato)cadium(II)]-μ-1,3-di-4-pyridylpropane]

**DOI:** 10.1107/S1600536809010447

**Published:** 2009-03-28

**Authors:** Yan Zhang, Yan Zhou, Lei Han

**Affiliations:** a Faculty of Materials Science & Chemical Engineering, Ningbo University, Ningbo, Zhejiang 315211, People’s Republic of China

## Abstract

In the title compound, [Cd(C_7_H_7_S)_2_(C_13_H_14_N_2_)]_*n*_, the unique Cd^II^ ion, located on a twofold rotation axis, is coordinated by two S atoms and two N atoms in a slightly distorted tetra­hedral environment. Symmetry-related Cd^II^ ions are linked *via* bridging 1,3-di-4-pyridylpropane ligands, forming a zig-zag chain-structure parallel to [001]. In the crystal structure, there are weak intra­chain π–π stacking inter­actions between benzene rings, with a centroid–centroid distance of 3.825 (7) Å, and pairs of chains are inter­digitated with respect to the 4-methyl­benzene­thiol­ate groups.

## Related literature

For background information on coordination polymers, see: James (2003 ▶); Wang *et al.* (2005 ▶); Cheng *et al.* (2007 ▶); Han & Zhou (2008 ▶). For information on the 1,3-bis­(4-pyrid­yl)propane ligand, see: Han *et al.* (2007 ▶); Carlucci *et al.* (2002 ▶). For the synthetic procedure, see: Dance *et al.* (1987 ▶).
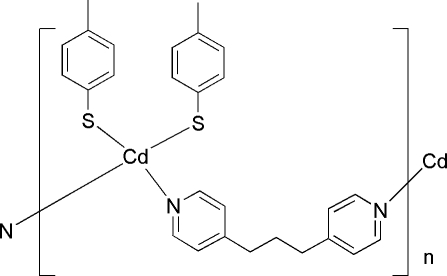


## Experimental

### 

#### Crystal data

[Cd(C_7_H_7_S)_2_(C_13_H_14_N_2_)]
*M* *_r_* = 557.03
Monoclinic, 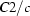

*a* = 11.922 (2) Å
*b* = 16.792 (3) Å
*c* = 12.862 (3) Å
β = 91.06 (3)°
*V* = 2574.5 (9) Å^3^
*Z* = 4
Mo *K*α radiation
μ = 1.03 mm^−1^
*T* = 298 K
0.45 × 0.25 × 0.18 mm

#### Data collection

Rigaku R-AXIS RAPID diffractometer
Absorption correction: multi-scan (*ABSCOR*; Higashi, 1995 ▶) *T*_min_ = 0.655, *T*_max_ = 0.837
12609 measured reflections
2948 independent reflections
2587 reflections with *I* > 2σ(*I*)
*R*_int_ = 0.046

#### Refinement

*R*[*F* ^2^ > 2σ(*F* ^2^)] = 0.036
*wR*(*F* ^2^) = 0.090
*S* = 1.09
2948 reflections
150 parameters
H atoms treated by a mixture of independent and constrained refinement
Δρ_max_ = 0.53 e Å^−3^
Δρ_min_ = −0.90 e Å^−3^

### 

Data collection: *RAPID-AUTO* (Rigaku, 1998 ▶); cell refinement: *RAPID-AUTO*; data reduction: *CrystalStructure* (Rigaku/MSC, 2004 ▶); program(s) used to solve structure: *SHELXS97* (Sheldrick, 2008 ▶); program(s) used to refine structure: *SHELXL97* (Sheldrick, 2008 ▶); molecular graphics: *SHELXTL* (Sheldrick, 2008 ▶) and *PLATON* (Spek, 2009 ▶); software used to prepare material for publication: *SHELXL97*.

## Supplementary Material

Crystal structure: contains datablocks I, global. DOI: 10.1107/S1600536809010447/lh2791sup1.cif

Structure factors: contains datablocks I. DOI: 10.1107/S1600536809010447/lh2791Isup2.hkl

Additional supplementary materials:  crystallographic information; 3D view; checkCIF report

## Figures and Tables

**Table:** 

Cd1—N1	2.320 (2)
Cd1—S1	2.4370 (9)

**Table:** 

N1—Cd1—N1^i^	93.43 (11)
N1—Cd1—S1	103.83 (7)
N1^i^—Cd1—S1	108.77 (7)
S1—Cd1—S1^i^	131.71 (4)

Symmetry code: (i) 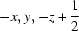.

**Fig. 1.**
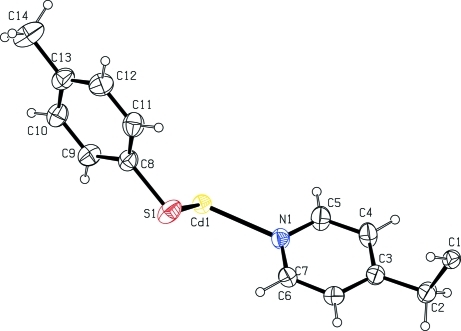
The asymmetric unit of the title compound showing 30% probability ellipsoids.

**Fig. 2.**
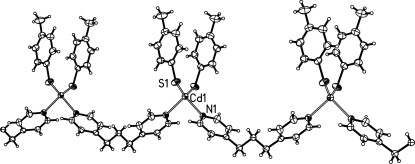
Part of the 1-D chain structure of title complex with 30% probability ellipsoids.

**Fig. 3.**
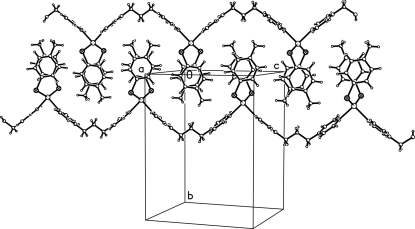
Part of the crsytal structutre showing two interdigitated 1-D chains.

## supplementary crystallographic information

### Comment

The search for new crystalline coordination polymers is fueled by the use of such materials in catalysis, separations, magnetism, and optoelectronics (James, 2003). Recently, interest has been devoted to the entanglement of 1D coordination polymers resulting in the architectures of an overall higher dimensionality (Wang *et al.*, 2005; Cheng *et al.*, 2007). The organic ligand, 1,3-bis(4-pyridyl)propane (bpp), is a long and flexible multi-functional linker, which can adopt different conformations with respect to the relative orientations of the CH_2_ groups (Han *et al.*, 2007; Carlucci *et al.*, 2002). We have reported a 2-D interwoven network entangled from zigzag chains using the bpp ligand as building unit (Han & Zhou, 2008). In an attempt to synthesize further interwoven networks  we have synthesized the one-dimensional polymer formed from Cd(SC_6_H_4_Me-4)_2_ and bpp and its crystal structure is reported herein.

The title compound, [Cd(SC_6_H_4_Me-4)_2_(bpp)]_n_, is a one-dimensional chain structure and the asymmetric unit is shown in Fig. 1. The unique Cd^II^ ion is coordinated by two S atoms and two N atoms adopting a slightly distorted tetrahedral coordination geometry. In the chain structure, there are weak *π**···**π* stacking interactions between two symmetry related benzene rings of the 4-methylbenzenethiolate groups, within the same chain. The centroid-to-centroid distance (Cg···Cg^i^) is 3.825 (7) Å (symmetry code: (i) -x, y, -z+1/2). The dihedral angle between two benzene rings is 3.2 (7)° (Fig. 2). Figure 3 shows part of the crystal structure of the title compound illustrating two interdigitated 1-D chains.

### Experimental

Cd(SC_6_H_4_Me-4)_2_ was synthesized according to the literature (Dance *et al.*, 1987). A mixture of Cd(SC_6_H_4_Me-4)_2 _(99.5 mg) and 1,3-bis(4-pyridyl)propane (50.1 mg) in DMF (6.0 g) solution was stirred for 30 min. The solution was allowed to stand at room temperature for 5 days. Colorless block crystals of the title complex were obtained and collected by filtration with a 30% yield.

### Refinement

The unique H atom on C1 was located in a difference map and refined freely. Other H atoms were positioned geometrically and allowed to ride on their respective parent atoms, with C—H = 0.93 Å and *U**_iso_*(H) = 1.2*U**_eq_*(C) for phenyl and pyridyl H atoms, C—H = 0.96 Å and *U**_iso_*(H) =1.5*U**_eq_*(C) for methyl, C—H = 0.97 Å and *U**_iso_*(H) = 1.2*U**_eq_*(C) for methylene.

### Crystal data

**Table:**

[Cd(C_7_H_7_S)_2_(C_13_H_14_N_2_)]	*F*(000) = 1136
*M**_r_* = 557.03	*D*_x_ = 1.437 Mg m^−^^3^
Monoclinic, *C*2/*c*	Mo *K*α radiation, λ = 0.71073 Å
Hall symbol: -C 2yc	Cell parameters from 1774 reflections
*a* = 11.922 (2) Å	θ = 3.2–27.5°
*b* = 16.792 (3) Å	µ = 1.03 mm^−^^1^
*c* = 12.862 (3) Å	*T* = 298 K
β = 91.06 (3)°	Block, colorless
*V* = 2574.5 (9) Å^3^	0.45 × 0.25 × 0.18 mm
*Z* = 4	

### Data collection

**Table:**

Rigaku R-AXIS RAPID diffractometer	2948 independent reflections
Radiation source: fine-focus sealed tube	2587 reflections with *I* > 2σ(*I*)
graphite	*R*_int_ = 0.046
Detector resolution: 0 pixels mm^-1^	θ_max_ = 27.5°, θ_min_ = 3.2°
ω scans	*h* = −15→15
Absorption correction: multi-scan (*ABSCOR*; Higashi, 1995)	*k* = −21→21
*T*_min_ = 0.655, *T*_max_ = 0.837	*l* = −16→14
12609 measured reflections	

### Refinement

**Table:**

Refinement on *F*^2^	Primary atom site location: structure-invariant direct methods
Least-squares matrix: full	Secondary atom site location: difference Fourier map
*R*[*F*^2^ > 2σ(*F*^2^)] = 0.036	Hydrogen site location: inferred from neighbouring sites
*wR*(*F*^2^) = 0.090	H atoms treated by a mixture of independent and constrained refinement
*S* = 1.09	*w* = 1/[σ^2^(*F*_o_^2^) + (0.0411*P*)^2^ + 2.0003*P*] where *P* = (*F*_o_^2^ + 2*F*_c_^2^)/3
2948 reflections	(Δ/σ)_max_ = 0.001
150 parameters	Δρ_max_ = 0.53 e Å^−^^3^
0 restraints	Δρ_min_ = −0.90 e Å^−^^3^

### Special details

**Table:**

Geometry. All esds (except the esd in the dihedral angle between two l.s. planes) are estimated using the full covariance matrix. The cell esds are taken into account individually in the estimation of esds in distances, angles and torsion angles; correlations between esds in cell parameters are only used when they are defined by crystal symmetry. An approximate (isotropic) treatment of cell esds is used for estimating esds involving l.s. planes.
Refinement. Refinement of F^2^ against ALL reflections. The weighted R-factor wR and goodness of fit S are based on F^2^, conventional R-factors R are based on F, with F set to zero for negative F^2^. The threshold expression of F^2^ > 2sigma(F^2^) is used only for calculating R-factors(gt) etc. and is not relevant to the choice of reflections for refinement. R-factors based on F^2^ are statistically about twice as large as those based on F, and R- factors based on ALL data will be even larger.

### Fractional atomic coordinates and isotropic or equivalent isotropic displacement parameters (Å^2^)

**Table:**

	*x*	*y*	*z*	*U*_iso_*/*U*_eq_	
Cd1	0.0000	0.295756 (15)	0.2500	0.04791 (11)	
S1	0.18408 (7)	0.35512 (5)	0.22501 (8)	0.0677 (2)	
N1	−0.0143 (2)	0.20104 (12)	0.11914 (17)	0.0485 (5)	
C1	0.0000	0.1037 (2)	−0.2500	0.0464 (8)	
H1	0.065 (2)	0.1374 (16)	−0.243 (2)	0.050 (8)*	
C2	−0.0111 (3)	0.05267 (16)	−0.1528 (2)	0.0579 (7)	
H2A	−0.0799	0.0219	−0.1579	0.070*	
H2B	0.0512	0.0156	−0.1485	0.070*	
C3	−0.0126 (3)	0.10235 (14)	−0.05565 (19)	0.0486 (6)	
C4	−0.1082 (3)	0.14257 (19)	−0.0269 (2)	0.0615 (7)	
H4A	−0.1741	0.1373	−0.0662	0.074*	
C5	−0.1060 (3)	0.19035 (19)	0.0598 (3)	0.0607 (8)	
H5A	−0.1716	0.2165	0.0779	0.073*	
C6	0.0779 (3)	0.16210 (17)	0.0914 (2)	0.0545 (7)	
H6A	0.1425	0.1682	0.1322	0.065*	
C7	0.0822 (3)	0.11328 (16)	0.0054 (2)	0.0551 (7)	
H7A	0.1488	0.0879	−0.0113	0.066*	
C8	0.1615 (2)	0.45217 (16)	0.2756 (2)	0.0511 (6)	
C9	0.1277 (3)	0.46512 (18)	0.3764 (2)	0.0589 (7)	
H9A	0.1152	0.4218	0.4197	0.071*	
C10	0.1120 (3)	0.54131 (18)	0.4140 (3)	0.0621 (7)	
H10A	0.0898	0.5482	0.4823	0.075*	
C11	0.1812 (3)	0.51806 (19)	0.2143 (2)	0.0643 (8)	
H11A	0.2056	0.5114	0.1467	0.077*	
C12	0.1646 (3)	0.59429 (19)	0.2530 (3)	0.0686 (9)	
H12A	0.1782	0.6379	0.2105	0.082*	
C13	0.1286 (3)	0.60699 (18)	0.3527 (3)	0.0626 (8)	
C14	0.1075 (4)	0.6900 (2)	0.3939 (4)	0.0969 (14)	
H14A	0.0836	0.6867	0.4646	0.145*	
H14B	0.0502	0.7152	0.3522	0.145*	
H14C	0.1754	0.7205	0.3909	0.145*	

### Atomic displacement parameters (Å^2^)

**Table:**

	*U*^11^	*U*^22^	*U*^33^	*U*^12^	*U*^13^	*U*^23^
Cd1	0.05340 (18)	0.04305 (16)	0.04712 (17)	0.000	−0.00332 (12)	0.000
S1	0.0523 (4)	0.0585 (4)	0.0927 (6)	−0.0030 (3)	0.0129 (4)	−0.0238 (4)
N1	0.0573 (14)	0.0480 (12)	0.0402 (11)	−0.0044 (10)	−0.0019 (10)	0.0000 (9)
C1	0.059 (2)	0.0403 (18)	0.0402 (18)	0.000	−0.0002 (17)	0.000
C2	0.089 (2)	0.0427 (13)	0.0418 (13)	−0.0028 (14)	−0.0010 (14)	0.0013 (11)
C3	0.0688 (17)	0.0394 (12)	0.0375 (12)	−0.0064 (11)	0.0005 (12)	0.0058 (9)
C4	0.0559 (17)	0.077 (2)	0.0511 (16)	−0.0052 (14)	−0.0066 (14)	−0.0122 (14)
C5	0.0511 (16)	0.076 (2)	0.0546 (17)	−0.0011 (14)	0.0006 (14)	−0.0139 (14)
C6	0.0584 (16)	0.0524 (15)	0.0521 (15)	0.0013 (13)	−0.0132 (13)	−0.0013 (12)
C7	0.0625 (17)	0.0493 (14)	0.0533 (16)	0.0088 (13)	−0.0032 (14)	0.0000 (12)
C8	0.0407 (13)	0.0525 (14)	0.0600 (16)	−0.0043 (11)	−0.0002 (12)	−0.0093 (12)
C9	0.0627 (18)	0.0524 (16)	0.0621 (17)	−0.0018 (13)	0.0115 (15)	0.0004 (13)
C10	0.0635 (18)	0.0632 (18)	0.0601 (17)	−0.0012 (14)	0.0091 (15)	−0.0109 (14)
C11	0.069 (2)	0.0673 (19)	0.0569 (17)	−0.0094 (15)	0.0043 (15)	−0.0023 (14)
C12	0.075 (2)	0.0543 (17)	0.076 (2)	−0.0070 (15)	0.0042 (18)	0.0067 (15)
C13	0.0561 (17)	0.0512 (16)	0.080 (2)	−0.0032 (13)	0.0031 (16)	−0.0113 (14)
C14	0.098 (3)	0.058 (2)	0.136 (4)	0.0021 (19)	0.023 (3)	−0.020 (2)

### Geometric parameters (Å, °)

**Table:**

Cd1—N1	2.320 (2)	C6—C7	1.379 (4)
Cd1—N1^i^	2.320 (2)	C6—H6A	0.9300
Cd1—S1	2.4370 (9)	C7—H7A	0.9300
Cd1—S1^i^	2.4370 (9)	C8—C11	1.381 (4)
S1—C8	1.777 (3)	C8—C9	1.383 (4)
N1—C6	1.333 (4)	C9—C10	1.381 (4)
N1—C5	1.334 (4)	C9—H9A	0.9300
C1—C2^ii^	1.523 (3)	C10—C13	1.372 (4)
C1—C2	1.523 (3)	C10—H10A	0.9300
C1—H1	0.97 (3)	C11—C12	1.389 (4)
C2—C3	1.503 (4)	C11—H11A	0.9300
C2—H2A	0.9700	C12—C13	1.376 (5)
C2—H2B	0.9700	C12—H12A	0.9300
C3—C7	1.377 (4)	C13—C14	1.513 (4)
C3—C4	1.381 (4)	C14—H14A	0.9600
C4—C5	1.373 (4)	C14—H14B	0.9600
C4—H4A	0.9300	C14—H14C	0.9600
C5—H5A	0.9300		
			
N1—Cd1—N1^i^	93.43 (11)	N1—C6—H6A	118.3
N1—Cd1—S1	103.83 (7)	C7—C6—H6A	118.3
N1^i^—Cd1—S1	108.77 (7)	C3—C7—C6	119.6 (3)
N1—Cd1—S1^i^	108.77 (7)	C3—C7—H7A	120.2
N1^i^—Cd1—S1^i^	103.83 (7)	C6—C7—H7A	120.2
S1—Cd1—S1^i^	131.71 (4)	C11—C8—C9	117.7 (3)
C8—S1—Cd1	100.62 (9)	C11—C8—S1	119.9 (2)
C6—N1—C5	116.9 (2)	C9—C8—S1	122.4 (2)
C6—N1—Cd1	118.72 (19)	C10—C9—C8	121.1 (3)
C5—N1—Cd1	123.8 (2)	C10—C9—H9A	119.4
C2^ii^—C1—C2	111.6 (3)	C8—C9—H9A	119.4
C2^ii^—C1—H1	109.0 (17)	C13—C10—C9	121.5 (3)
C2—C1—H1	109.5 (16)	C13—C10—H10A	119.3
C3—C2—C1	111.9 (2)	C9—C10—H10A	119.3
C3—C2—H2A	109.2	C8—C11—C12	120.5 (3)
C1—C2—H2A	109.2	C8—C11—H11A	119.8
C3—C2—H2B	109.2	C12—C11—H11A	119.8
C1—C2—H2B	109.2	C13—C12—C11	121.7 (3)
H2A—C2—H2B	107.9	C13—C12—H12A	119.1
C7—C3—C4	117.1 (2)	C11—C12—H12A	119.1
C7—C3—C2	121.7 (3)	C10—C13—C12	117.5 (3)
C4—C3—C2	121.1 (3)	C10—C13—C14	120.9 (3)
C5—C4—C3	120.0 (3)	C12—C13—C14	121.7 (3)
C5—C4—H4A	120.0	C13—C14—H14A	109.5
C3—C4—H4A	120.0	C13—C14—H14B	109.5
N1—C5—C4	123.1 (3)	H14A—C14—H14B	109.5
N1—C5—H5A	118.5	C13—C14—H14C	109.5
C4—C5—H5A	118.5	H14A—C14—H14C	109.5
N1—C6—C7	123.3 (3)	H14B—C14—H14C	109.5

Symmetry codes: (i) −*x*, *y*, −*z*+1/2; (ii) −*x*, *y*, −*z*−1/2.
